# Parent attention‐orienting behavior is associated with neural entropy in infancy

**DOI:** 10.1111/infa.12595

**Published:** 2024-04-27

**Authors:** Cabell L. Williams, Allison R. Belkowitz, Madelyn G. Nance, Emily T. Mortman, Sonie Bae, Sheher‐Bano Ahmed, Meghan H. Puglia

**Affiliations:** ^1^ Department of Psychology University of Virginia Charlottesville Virginia USA; ^2^ Department of Neurology University of Virginia Charlottesville Virginia USA; ^3^ Department of Neuroscience Smith College Northampton Massachusetts USA

## Abstract

Parents use joint attention to direct infants to environmental stimuli. We hypothesized that infants whose parents provide more bids for joint attention will display a more complex neural response when viewing social scenes. Sixty‐one 8‐month‐old infants underwent electroencephalography (EEG) while viewing videos of joint‐ and parallel‐play and participated in a free play interaction. EEG data was analyzed using multiscale entropy, which quantifies neural variability. Free play interactions assessed parent alternating gaze, a behavioral mechanism for directing attention to environmental cues. We found a significant positive association between parent alternating gaze and neural entropy in frontal and central regions. These results suggest a relationship between parent behavior and infant neural mechanisms that regulate social attention, underlying the importance of parental cues in forming neural networks.

## 1 INTRODUCTION

Caregiver social cues are critical for enabling infants to engage with their environment (Moore & Dunham, 1995; Tomasello, 1995). Joint attention is a preverbal referential behavior by which individuals follow or direct another's attention (Bruner, 1974) that emerges around 6 months (Butterworth & Jarrett, 1991). Joint attention has been associated with consolidation of social information (Mundy & Gomes, 1998), language acquisition (Mundy & Gomes, 1998; Salo et al., 2018; Tomasello & Farrar, 1986), greater visual attention to a partner (Striano & Stahl, 2005), proficiency in culturally normative behavior (Bruner, 1974), and educational success (Mundy & Newell, 2007). A key parent‐directed joint attention behavior occurs when parents alternate gaze between the infant and an object (Bakeman & Adamson, 1984). The effects of parent‐, rather than infant‐directed joint attention (e.g., pointing to direct caregiver attention [Carpenter et al., 1998]), on infant neural responses to social information has not been explored. This research aims to assess how the early caregiving environment impacts infant social‐attentional brain function.

Infancy is a sensitive period of development characterized by neural plasticity. Infant brains are highly susceptible to experiential neural rewiring (Bennett et al., 2018; Kolb et al., 2017), resulting in greater functional variability and neurological complexity (McIntosh et al., 2010). Neural entropy—a measure of functional variability and complexity—has been associated with information exchange and synchrony between neurons (Mišić et al., 2015; Shew et al., 2009, 2011), and may facilitate attention to and detection of important environmental signals. We hypothesized that infants whose parents provide a more socially‐oriented environment (i.e., more joint attention bids through parent alternating gaze) will demonstrate increased neural entropy in regions supporting attention orienting (Hopfinger et al., 2000) and social perception (Grossmann, 2015) when viewing children engaging in joint‐play compared to parallel‐play.

## 2 METHODS

### 2.1 Participants

Sixty‐nine infants were recruited from the University of Virginia's (UVA) hospital and the greater Charlottesville area for a longitudinal study at birth, 4, 8, 12 and 16 months. Given that joint attention emerges around 6 months (Butterworth & Jarrett, 1991), we assessed neural and behavioral data from the 8‐month visit (*M* = 248.56 days, *SD* = 13.22). Infants completed a free play interaction with their parent and underwent electroencephalography (EEG). Parents received $50 per visit for participating. This study was conducted in accordance with the Declaration of Helsinki, with written informed consent obtained from each infant's parent prior to data collection. All procedures involving human subjects were approved by the University of Virginia's Institutional Review Board. Eight participants were excluded due to insufficient artifact‐free EEG data (see EEG Acquisition and Preprocessing). The final dataset consisted of 61 (33 female) infants.

### 2.2 Infant behavior

The parent and infant underwent a 5‐min videotaped naturalistic free play interaction in which they were instructed to play as they would at home. The first minute of each video was discarded to allow the dyad to acclimate to the environment. Videos were then coded using INTERACT software (Mangold) by a research assistant blind to the hypothesis for duration of parent alternating gaze, defined as the parent directing visual attention to their infant, then to an object, and back again for maximum 2‐s per turn. Twenty‐five percent of randomly selected videos were coded by a secondary coder to establish interrater reliability (two‐way random effects model, *r* = 0.81) (Koo & Li, 2016).

### 2.3 EEG Acquisition and Preprocessing

Infants, placed on their parent's lap, viewed 16‐s videos of two children engaging in joint‐ or parallel‐play while undergoing EEG. Video order was pseudorandomized such that no child pair was shown back‐to‐back, and no play viewing condition (joint/parallel) repeated more than twice. The next video began once the infant fixated on an attention‐getter at screen center for 500 ms, as measured by a Pro Spectrum (Tobii) eye tracker. The videos continued until the infant failed to orient to the screen for six consecutive trials or all 16 trials were completed. Visual attention to the paradigm was assessed via eye tracking, video recording, and experimenter observation. The videos were presented using PsychToolBox v3.0.14 (Brainard, 1997) for MATLAB (Mathworks). Parents were instructed not to interact with the infant and wore sunglasses to prevent the eye tracker from registering their gaze.

**FIGURE 1:**
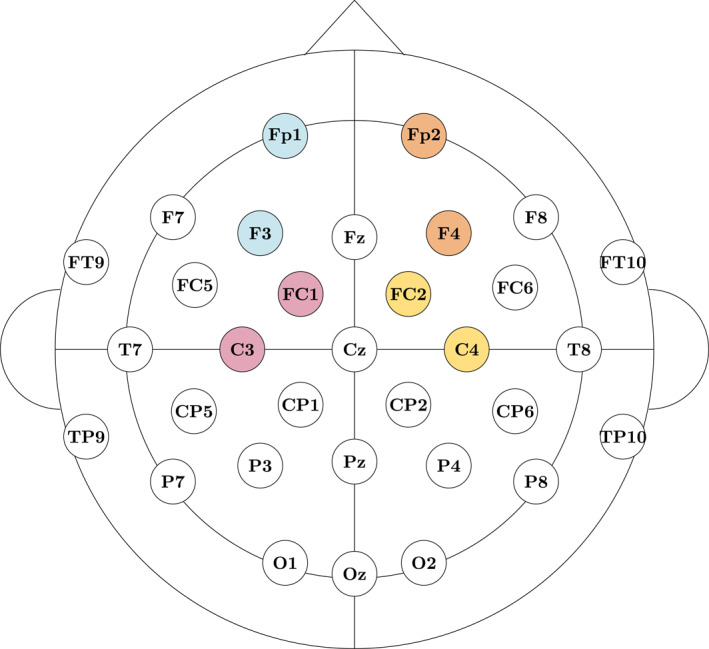
Infant EEG electrode montage. EEG data from 3 to 8 Hz was averaged together to create the following regions of interest: left frontal: Fp1 and F3 (blue); right frontal Fp2 and F4 (orange); left central: FC1 and C3 (red); right central: FC2 and C4 (yellow). All other scalp electrodes were grouped as a comparison region of interest. EEG, electroencephalography.

EEG data was collected using 32 Ag/AgCl active actiCAP slim electrodes (Brain Products GmbH) fastened to an elastic cap following the 10–20 electrode placement system (Figure 1). EEG signals were amplified using a BrainAmp DC Amplifier and recorded with BrainVision Recorder software at 5000 Hz with an online reference to FCz, and an online band‐pass filter between 0.01 and 1000 Hz.

EEG preprocessing was completed using the Automated Preprocessing Pipe‐Line for the Estimation of Scale‐wise Entropy from EEG Data (APPLESEED) (Puglia et al., 2022), a standardized EEG preprocessing and entropy estimation pipeline for pediatric EEG data implemented through EEGLab (Delorme & Makeig, 2004). We down sampled the data to 500 Hz, band pass filtered from 0.1 to 100 Hz, and removed 60 Hz sinusoidal noise using CleanLine (Cleanline, 2019/2023). Sixteen 1‐s epochs were extracted from each video. Channels exceeding 500 μV in greater than 50% of epochs were spherically interpolated. Epochs contaminated with excessive amplitude standard deviations (>80 μV) within a 200‐ms sliding window were discarded. The data was re‐referenced to the average of all scalp electrodes. Because the number of data points influences the reliability of multiscale entropy (MSE) estimates (Grandy et al., 2016), it is critical that MSE is computed from an equivalent number of data points for each participant. Therefore, the 10 trials for each viewing condition (DeBoer et al., 2007; Stets et al., 2012) with a total global field power (GFP) closest to the median GFP for each participant were selected for downstream analysis. Eight participants had fewer than 10 artifact‐free trials in each viewing condition and were excluded from subsequent analysis. The final dataset consisted of 61 infants.

### 2.4 Analysis

MSE quantifies signal variability and irregularity across time scales (Costa et al., 2002) by measuring iterative patterns within a time series and assigning low values to predictable patterns and high values to unpredictable patterns. Neural systems operate on multiple timescales simultaneously, necessitating the use of a multiscale approach for measuring neural signal variability. MSE analysis was computed using APPLESEED (Puglia et al., 2022) with a pattern length *m* = 2 and similarity criterion *r* = 0.5. Critically, this pipeline uses a modified MSE algorithm that recomputes *r* at each scale (Nikulin & Brismar, 2004) and computes MSE across discontinuous segments (Grandy et al., 2016) enabling entropy estimates at more fine‐grained time scales. Entropy estimates within the 3–8 Hz range—the predominant frequency in 8‐month‐old infants (Saby & Marshall, 2012)—were averaged together within four regions of interest (ROIs): left frontal (Fp1, F3), right frontal (Fp2, F4), left central (FC1, C3), and right central (FC2, C4)—selected as regions implicated in attention orienting (Hopfinger et al., 2000) and social perception (Grossmann, 2015). All other electrodes were grouped as a comparison ROI (Figure 1).

Statistics were run in RStudio v2022.12.0 (RStudio Team, 2020). A general linear model assessed the association between parent alternating gaze and MSE with viewing condition (joint vs. parallel) and ROI (left frontal, right frontal, left central right central, other) included as covariates. Parent alternating gaze was *z*‐score transformed; the square root of MSE was calculated to validate the assumption of normally distributed residuals. Outlier data points were identified as those for which the absolute value of the median standardized residuals was >3. Three outliers in the joint‐play condition (1 left frontal, 1 right frontal, and 1 right central) were removed. Then, we tested for data points with undue influence on the model using Cook's distance (D) > 1 (Cook & Weisberg, 1982) as criteria. No influential points (all Ds < 0.02) were detected. Post‐hoc comparisons of estimated marginal means were used to explore differences across investigated ROIs via the Emmeans R package (Lenth R, 2018).

## 3 RESULTS

**FIGURE 2:**
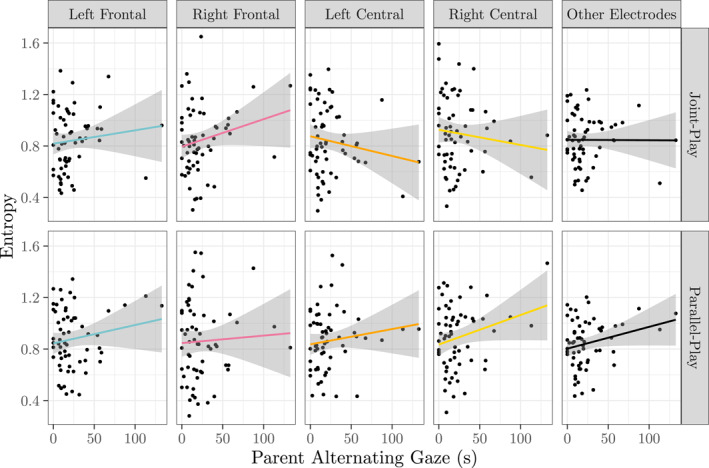
Association between parent alternating gaze and MSE. MSE was computed on infant electroencephalography data from 3 to 8 Hz for five regions of interest (vertical panels) over two play viewing conditions (horizontal panels). Parent alternating gaze is significantly associated with MSE (*β* = 0.013, *p* = −0.042). While there is no significant effect of play condition (*β* = 0.0087 *p* = 0.512) or region on interest (all ps > 0.361), there is a trending shift from a positive association in the frontal lobe (blue, red) to a negative association in the central lobe (orange, yellow) specific to the social processing (joint play viewing) condition (top panel). MSE, multiscale entropy.

We found a significant positive effect of parent alternating gaze (*β* = 0.013, *p* = 0.042) on entropy, but no effect of viewing condition (*β* = 0.007, *p* = 0.512) or ROI (all ps > 0.361). While post‐hoc comparisons revealed no statistically significant differences between ROIs (all ps > 0.636), a visual examination of the plotted associations revealed trending associations among our considered variables (Figure 2). Within the joint‐play viewing condition, we identified a trending positive association between parent alternating gaze and infant neural entropy in frontal regions, whereas we identified a trending negative association between these variables in central and other brain regions. However, during parallel‐play viewing, parent alternating gaze was positively associated with infant neural entropy in all brain regions.

## 4 DISCUSSION

This study assessed parent gaze orienting as an important factor in the modulation of infant social attention through increased neural variability. Our results showed a positive association between parent alternating gaze and neural entropy in 8‐month‐olds. At 8 months, infants are developing attentional control (Stroganova et al., 1998), a process facilitated by parent gaze cues which helps infants regulate attention to relevant external stimuli (Salley et al., 2016). This skill enables increased mutual gaze between infants and parents, which is key for developing sociality (e.g., sense of self, emotion regulation) (Niedźwiecka et al., 2018).

Our finding that parent alternating gaze is associated with increased neural entropy aligns with current literature surrounding the functionality of signal variability. Increased entropy represents a more complex, mature system capable of exploring varying brain states (Wang et al., 2018). A positive association between parent gaze cues and neural entropy infers that the infant finds social information maximally salient (Puglia et al., 2018) and may reflect accelerated development (Paterson et al., 2006).

We found trending effects of viewing condition (joint‐vs. parallel‐play) on the association between MSE and parent behavior across central and frontal cortices. The association trends positive in frontal and negative in central regions for the joint‐play viewing condition only, suggesting that 8‐month‐old infants discriminate social interactions within brain regions specifically involved with attention regulation (Hopfinger et al., 2000) and social information processing (Grossmann, 2015).

While significant, the effect sizes and variance explained were low. Future investigations should replicate these results with a larger sample, consider additional social influences, and longitudinally assess associations between parent behavior and infant social‐attentional development (Cleveland & Striano, 2007).

### 4.1 Conclusions

Neural plasticity occurs as environmental factors shape infant neurodevelopment. Parent gaze is an important cue that facilitates an infant's ability to perceive and interpret external stimuli, which is critical to neural network development underlying social skills. We demonstrated that parent‐directed social orienting cues modulate neural variability reflective of attentional states during social processing. This research highlights the importance of early caregiving on emerging social attentional skills in infancy.

## Data Availability

The data that support the findings of this study are available from the corresponding author upon reasonable request.

## References

[infa12595-bib-0001] Bakeman, R. , & Adamson, L. B. (1984). Coordinating attention to people and objects in mother–infant and peer–infant interaction. Child Development, 55(4), 1278–1289. 10.2307/1129997 6488956

[infa12595-bib-0002] Bennett, S. H. , Kirby, A. J. , & Finnerty, G. T. (2018). Rewiring the connectome: Evidence and effects. Neuroscience and Biobehavioral Reviews, 88, 51–62. 10.1016/j.neubiorev.2018.03.001 29540321 PMC5903872

[infa12595-bib-0003] Brainard, D. (1997). The psychophysics toolbox. Spatial Vision, 10(4), 433–436. 10.1163/156856897x00357 9176952

[infa12595-bib-0004] Bruner, J. S. (1974). From communication to language—a psychological perspective. Cognition, 3(3), 255–287. 10.1016/0010-0277(74)90012-2

[infa12595-bib-0005] Butterworth, G. , & Jarrett, N. (1991). What minds have in common is space: Spatial mechanisms serving joint visual attention in infancy. British Journal of Developmental Psychology, 9(1), 55–72. 10.1111/j.2044-835x.1991.tb00862.x

[infa12595-bib-0006] Carpenter, M. , Nagell, K. , Tomasello, M. , Butterworth, G. , & Moore, C. (1998). Social cognition, joint attention, and communicative competence from 9 to 15 Months of age. Monographs of the Society for Research in Child Development, 63(4), i–174. 10.2307/1166214 9835078

[infa12595-bib-0007] Cleanline . (2023). [MATLAB]. Swartz center for computational neuroscience. (Original work published 2019) https://github.com/sccn/cleanline

[infa12595-bib-0008] Cleveland, A. , & Striano, T. (2007). The effects of joint attention on object processing in 4‐ and 9‐month‐old infants. Infant Behavior and Development, 30(3), 499–504. 10.1016/j.infbeh.2006.10.009 17610957

[infa12595-bib-0009] Cook, R. D. , & Weisberg, S. (1982). Residuals and influence in regression. Chapman and Hall. http://conservancy.umn.edu/handle/11299/37076

[infa12595-bib-0010] Costa, M. , Goldberger, A. L. , & Peng, C‐K. (2002). Multiscale entropy analysis of complex physiologic time series. Physical Review Letters, 89(6), 068102. 10.1103/PhysRevLett.89.068102 12190613

[infa12595-bib-0011] DeBoer, T. , Scott, L. S. , & Nelson, C. A. (2007). Methods for acquiring and analyzing infant event‐related potentials. In Infant EEG and event‐related potentials (pp. 5–37). Psychology Press.

[infa12595-bib-0012] Delorme, A. , & Makeig, S. (2004). Eeglab: An open source toolbox for analysis of single‐trial EEG dynamics including independent component analysis. Journal of Neuroscience Methods, 134(1), 9–21. 10.1016/j.jneumeth.2003.10.009 15102499

[infa12595-bib-0013] Grandy, T. H. , Garrett, D. D. , Schmiedek, F. , & Werkle‐Bergner, M. (2016). On the estimation of brain signal entropy from sparse neuroimaging data. Scientific Reports, 6(1), 23073. Article 1. 10.1038/srep23073 27020961 PMC4810375

[infa12595-bib-0014] Grossmann, T. (2015). The development of social brain functions in infancy. Psychological Bulletin, 141(6), 1266–1287. 10.1037/bul0000002 25984728

[infa12595-bib-0015] Hopfinger, J. B. , Buonocore, M. H. , & Mangun, G. R. (2000). The neural mechanisms of top‐down attentional control. Nature Neuroscience, 3(3), 284–291. Article 3. 10.1038/72999 10700262

[infa12595-bib-0016] Kolb, B. , Harker, A. , & Gibb, R. (2017). Principles of plasticity in the developing brain. Developmental Medicine and Child Neurology, 59(12), 1218–1223. 10.1111/dmcn.13546 28901550

[infa12595-bib-0017] Koo, T. K. , & Li, M. Y. (2016). A guideline of selecting and reporting intraclass correlation coefficients for reliability research. Journal of Chiropractic Medicine, 15(2), 155–163. 10.1016/j.jcm.2016.02.012 27330520 PMC4913118

[infa12595-bib-0018] Lenth, R. (2018). Emmeans: Estimated marginal means. AKA Least‐Squares Means, 1(7). https://CRAN.R‐project.org/package=emmeans

[infa12595-bib-0019] McIntosh, A. R. , Kovacevic, N. , Lippe, S. , Garrett, D. , Grady, C. , & Jirsa, V. (2010). The development of a noisy brain. Archives Italiennes de Biologie, 148(3), 323–337. 21175017

[infa12595-bib-0020] Mišić, B. , Doesburg, S. M. , Fatima, Z. , Vidal, J. , Vakorin, V. A. , Taylor, M. J. , & McIntosh, A. R. (2015). Coordinated information generation and mental flexibility: Large‐scale network disruption in children with autism. Cerebral Cortex, 25(9), 2815–2827. 10.1093/cercor/bhu082 24770713 PMC4537433

[infa12595-bib-0021] Moore, C. , & Dunham, P. J. (1995). Joint attention: Its origins and role in development. https://philpapers.org/rec/MOOJAI‐2

[infa12595-bib-0022] Mundy, P. , & Gomes, A. (1998). Individual differences in joint attention skill development in the second year. Infant Behavior and Development, 21(3), 469–482. 10.1016/S0163-6383(98)90020-0

[infa12595-bib-0023] Mundy, P. , & Newell, L. (2007). Attention, joint attention, and social cognition. Current Directions in Psychological Science, 16(5), 269–274. 10.1111/j.1467-8721.2007.00518.x 19343102 PMC2663908

[infa12595-bib-0024] Niedźwiecka, A. , Ramotowska, S. , & Tomalski, P. (2018). Mutual gaze during early mother‐infant interactions promotes attention control development. Child Development, 89(6), 2230–2244. 10.1111/cdev.12830 28510324

[infa12595-bib-0025] Nikulin, V. V. , & Brismar, T. (2004). Comment on ``Multiscale entropy analysis of complex physiologic time series. Physical Review Letters, 92(8), 089803. 10.1103/PhysRevLett.92.089803 14995828

[infa12595-bib-0026] Paterson, S. J. , Heim, S. , Friedman, J. T. , Choudhury, N. , & Benasich, A. A. (2006). Development of structure and function in the infant brain: Implications for cognition, language and social behaviour. Neuroscience & Biobehavioral Reviews, 30(8), 1087–1105. 10.1016/j.neubiorev.2006.05.001 16890291 PMC1933387

[infa12595-bib-0027] Puglia, M. H. , Connelly, J. J. , & Morris, J. P. (2018). Epigenetic regulation of the oxytocin receptor is associated with neural response during selective social attention. Translational Psychiatry, 8(1), 1–10. 10.1038/s41398-018-0159-x 29907738 PMC6003910

[infa12595-bib-0028] Puglia, M. H. , Slobin, J. S. , & Williams, C. L. (2022). The automated preprocessing pipe‐line for the estimation of scale‐wise entropy from EEG data (APPLESEED): Development and validation for use in pediatric populations. Developmental Cognitive Neuroscience, 58, 101163. 10.1016/j.dcn.2022.101163 36270100 PMC9586850

[infa12595-bib-0029] RStudio Team . (2020). RStudio: Integrated development for R. RStudio. http://www.rstudio.com

[infa12595-bib-0030] Saby, J. , & Marshall, P. (2012). The utility of EEG band power analysis in the study of infancy and early childhood—PMC. Developmental Neuropsychology, 37(3), 253–273. 10.1080/87565641.2011.614663 22545661 PMC3347767

[infa12595-bib-0031] Salley, B. , Sheinkopf, S. J. , Neal‐Beevers, A. R. , Tenenbaum, E. J. , Miller‐Loncar, C. L. , Tronick, E. , Lagasse, L. L. , Shankaran, S. , Bada, H. , Bauer, C. , Whitaker, T. , Hammond, J. , & Lester, B. M. (2016). Infants’ early visual attention and social engagement as developmental precursors to joint attention. Developmental Psychology, 52(11), 1721–1731. 10.1037/dev0000205 27786527 PMC5117646

[infa12595-bib-0032] Salo, V. C. , Rowe, M. L. , & Reeb‐Sutherland, B. C. (2018). Exploring infant gesture and joint attention as related constructs and as predictors of later language. Infancy, 23(3), 432–452. 10.1111/infa.12229 29725273 PMC5927593

[infa12595-bib-0033] Shew, W. L. , Yang, H. , Petermann, T. , Roy, R. , & Plenz, D. (2009). Neuronal avalanches imply maximum dynamic range in cortical networks at criticality. Journal of Neuroscience: The Official Journal of the Society for Neuroscience, 29(49), 15595–15600. 10.1523/JNEUROSCI.3864-09.2009 20007483 PMC3862241

[infa12595-bib-0034] Shew, W. L. , Yang, H. , Yu, S. , Roy, R. , & Plenz, D. (2011). Information capacity and transmission are maximized in balanced cortical networks with neuronal avalanches. Journal of Neuroscience: The Official Journal of the Society for Neuroscience, 31(1), 55–63. 10.1523/JNEUROSCI.4637-10.2011 21209189 PMC3082868

[infa12595-bib-0035] Stets, M. , Stahl, D. , & Reid, V. M. (2012). A meta‐analysis investigating factors underlying attrition rates in infant ERP studies. Developmental Neuropsychology, 37(3), 226–252. 10.1080/87565641.2012.654867 22545660

[infa12595-bib-0036] Striano, T. , & Stahl, D. (2005). Sensitivity to triadic attention in early infancy. Developmental Science, 8(4), 333–343. 10.1111/j.1467-7687.2005.00421.x 15985067

[infa12595-bib-0037] Stroganova, T. A. , Orekhova, E. V. , & Posikera, I. N. (1998). Externally and internally controlled attention in infants: An EEG study. International Journal of Psychophysiology: Official Journal of the International Organization of Psychophysiology, 30(3), 339–351. 10.1016/s0167-8760(98)00026-9 9834890

[infa12595-bib-0038] Tomasello, M. (1995). Joint attention as social cognition. In Joint attention: Its origins and role in development (pp. 103–130). Lawrence Erlbaum Associates, Inc.

[infa12595-bib-0039] Tomasello, M. , & Farrar, M. J. (1986). Joint attention and early language. Child Development, 57(6), 1454–1463. 10.2307/1130423 3802971

[infa12595-bib-0040] Wang, D. J. J. , Jann, K. , Fan, C. , Qiao, Y. , Zang, Y‐F. , Lu, H. , & Yang, Y. (2018). Neurophysiological basis of multi‐scale entropy of brain complexity and its relationship with functional connectivity. Frontiers in Neuroscience, 12, 352. 10.3389/fnins.2018.00352 29896081 PMC5986880

